# Remaining Questions about Clinical Variola Major

**DOI:** 10.3201/eid1704.101960

**Published:** 2011-04

**Authors:** J. Michael Lane

**Affiliations:** Author affiliation: Emory University School of Medicine, Atlanta, Georgia, USA (retired)

**Keywords:** Smallpox, viruses, vaccinia, smallpox vaccines, variola major, antiviral drugs, bioterrorism and preparedness, policy review

## Abstract

After the recent summary of World Health Organization–authorized research on smallpox, several clinical issues remain. This policy review addresses whether early hemorrhagic smallpox is disseminated intravascular coagulation and speculates about the cause of the high mortality rate among pregnant women and whether ocular smallpox is partly the result of trachoma or vitamin A deficiency. The joint destruction common in children with smallpox might be prevented by antiviral drugs, but intraarticular infusion of antiviral drugs is unprecedented. Development of highly effective antiviral drugs against smallpox raises the issue of whether postexposure vaccination can be performed without interference by an antiviral drug. Clinicians should consider whether patients with smallpox should be admitted to general hospitals. Although an adequate supply of second-generation smallpox vaccine exists in the United States, its use is unclear. Finally, political and ethical forces suggest that destruction of the remaining stocks of live smallpox virus is now appropriate.

After the World Health Organization (WHO) declared smallpox eradicated in 1980, several problems remained concerning the disease and its causative virus, variola major virus*.* These problems included high rates of adverse events associated with most strains of vaccinia virus; our inadequate understanding of the pathophysiology of smallpox; lack of a good animal model of the disease; difficulty of rapid laboratory diagnosis, including the poor ability of most standard laboratory tests to distinguish between orthopoxviruses; lack of an effective antiviral drug; and rudimentary knowledge about the genetic makeup of the virus.

Although many observers wished to destroy the remaining stocks of variola major virus in 1980, several respected researchers wanted to use the live virus to help answer some of these remaining questions. In 1999, WHO agreed to a research agenda, with oversight by a WHO committee, to continue research with live variola major virus until substantial progress was made on these questions. The WHO oversight committee has now declared that satisfactory (if in some areas imperfect) progress has been made toward developing improved vaccines, better laboratory diagnostics, a reasonable nonhuman primate animal model, effective antiviral drugs, and good understanding of the genetics of orthopoxviruses. Reports of this progress have been published (*1**–**5*).

Despite this impressive progress, clinicians have been left with several unanswered questions. However, these questions may never be answered because we hope that there will never be another patient with classical smallpox, and the best nonhuman primate model does not perfectly reproduce clinical smallpox.

## Is Early Hemorrhagic Smallpox Disseminated Intravascular Coagulation?

Smallpox was eradicated before cases of early hemorrhagic disease could be studied in modern clinical settings. This disease had all the hallmarks of disseminated intravascular coagulation (DIC). Patients had widespread hemorrhaging in the skin during the early septic phase, usually before a rash developed. Bleeding occurred from multiple orifices. Although necropsy evidence is minimal, internal bleeding likely affected many organs. Results of studies of bleeding time, clotting time, and tourniquet tests in patients with early hemorrhagic smallpox were consistent with what might be expected with DIC (*6*). In addition, patients experienced high fever, cardiovascular collapse, and other clinical signs that we now associate with the cytokine cascade. Treating DIC remains difficult, but the case-fatality rate of early hemorrhagic smallpox approached 100%, and it might be reduced if treatment for DIC is instituted.

## Are High Case-Fatality Rates in Unvaccinated Pregnant Women and Fetal Wastage a Result of Immune Suppression?

Evidence has shown that the death rate from smallpox among pregnant women was extraordinarily high. Pregnant women had a higher rate of hemorrhagic disease than did other adults. Approximately 16% of cases in unvaccinated pregnant women were early hemorrhagic smallpox versus ≈1% in nonpregnant women and adult males. The case-fatality rate in unvaccinated pregnant women approached 70%. Fetal wastage approached 80% (*6**–**9*).

We now know that a normal pregnancy includes a modest transient immune deficit, particularly suppression of Th1 and Tc cells (*9*). If therapeutic interventions were available that could assist the immune system during infection with smallpox virus, the case-fatality rate in pregnant women might be reduced considerably.

## Are Ocular Variola and Its Resulting Blindness a Result of Trachoma?

Ocular variola was fairly common in the Asian subcontinent. Severe conjunctivitis was common in patients with smallpox, and corneal lesions developed in ≈7% of unvaccinated patients (*6*). Dixon reported that corneal lesions were most common in North Africa in patients with trachoma (*10*). Actual pocks occurred in vascularized parts of the conjunctiva or sclera in which pannus occurred. Obvious protein–calorie malnutrition also seemed to be a risk factor. Blood vessels in the conjunctiva and sclera characteristic of trachoma or other types of serious conjunctivitis in tropical areas enabled variola virions to be deposited on parts of the sclera that are usually avascular.

We cannot say with confidence that ocular variola was considerably more common in areas where trachoma or vitamin A deficiency was rare because rates of this devastating complication are not well documented in Europe and the United States; however, ocular variola certainly occurred. If trachoma is a predisposing condition, ocular variola may be rare in Western industrialized nations if smallpox reappears.

## What is the Mechanism for Joint Destruction by Variola Major?

Variola major resulted in destruction of large joints, particularly of the elbows and knees, in ≈2% of unvaccinated children. It seems likely that that this joint destruction was caused by infection of the joint space or compromise of the blood supply by a viral arteritis, rather than by an immune-mediated arthropathy (*11*). Could ST-246 or CMX-001 be injected directly into the joint space, and if so would it help? If the mechanism is predominately poor blood flow secondary to an arteritis, an antiviral drug might not eliminate it. Direct injection of an antiviral drug into the joint spaces might be useful if we could agree on clinical indicators of joint infection that would induce such a therapeutic approach.

## Will Vaccination during the Early Incubation Period, with or without Antiviral Drugs, Prevent Disease?

It was unethical, and probably logistically impossible, to conduct controlled field trials of vaccination at various periods into the incubation period during the many years that smallpox was being eliminated by using vigorous surveillance and vaccination of immediate contacts. A Delphi technique poll of experienced field workers concluded that most experts believed that vaccination within 4 days of exposure would prevent smallpox (*12*). Analysis of old data from the United Kingdom showed that good protection resulted from postexposure vaccination (*13*). The dynamics of development of humoral and cellular immunity after vaccination also suggest that vaccination within 3 days after exposure would be successful (*14*).

ST-246 seems to be an effective antiviral drug (*2**,**15*). Preliminary animal data and limited human data suggest that giving ST-246 with vaccination greatly reduces the clinical manifestations of vaccinia but does not impede development of cellular or humoral immunity (*15*). However, it seems counterintuitive to give a drug that virtually eliminates poxvirus replication and vaccinia virus unless we can be sure it will not reduce the effectiveness of vaccination.

## Where Should Patients with New Cases of Smallpox Be Treated?

Smallpox was a nosocomial disease (*16**,**17*). Often a patient who initially had no diagnosis was imperfectly isolated, despite having a high fever. The virus spread to other patients and to medical staff by close personal contact. The disease was considered by many experts to be most common in caregivers. In modern practice, 2 points should be considered when framing strategies for the use of hospitals during outbreak control. The first consideration is the use of therapies that may, although unproven, be of considerable value in reducing the case-fatality rate. These therapies include newly developed antiviral drugs, pressor therapy for shock, treatment for DIC, and efforts to control the cytokine cascade. The second consideration is the presence in modern hospitals of patients with HIV, iatrogenic immune suppression, and atopic dermatitis. These patients might become severely ill if exposed to smallpox virus. Their immune conditions may make vaccination difficult or dangerous if they are exposed to smallpox virus.

Evidence has shown that protocols for isolation of patients with fever and an undiagnosed rash are not rigidly followed in many general hospitals (*18*). It might be better to bring medical care to patients in a remote location (e.g., motel or defunct hospital) than to risk spread of the disease in a hospital. Fairly sophisticated medical care can now be given at home or in remote locations (*19*).

## What Would Be the Characteristics of a Smallpox Virus Created by Bioengineering?

Recreation of the smallpox virus from published genetic sequences (www.poxvirus.org) is theoretically possible. Inserting minor modifications into the genome of a well-characterized strain of vaccinia virus should be even easier. Orthopoxviruses are large, stable, DNA viruses that are fairly easy to manipulate genetically (*3**,**20**,**21*). Technologies needed for creating live poxviruses from a variety of genetic fragments are readily available (*3**,**20*). Some practical issues would be involved in such laboratory recreation, but a modern well-equipped viral genetics laboratory would have minimal difficulties.

The Soviet Union allegedly inserted genes from other pathogens into variola major virus (*21*). Researchers working with mousepox virus have created a recombinant virus capable of escaping the effects of prior immunization with vaccinia virus (*22*).

Such work has obvious ethical problems, but would creating live smallpox viruses be something terrorists would really want to do? Although most Western nations have mechanisms for controlling smallpox outbreaks, most third-world nations do not. The experience with severe acute respiratory syndrome showed that even in the absence of a vaccine or antimicrobial drug, diseases spread by respiratory secretions can be controlled by vigorous identification and isolation of patients (*23**,**24*). If widespread transmission occurs of a newly created or genetically modified variola major virus, it is highly likely that it would spread to third-world nations, including homelands of terrorists. The resulting devastation would create a major public relations setback to terrorists. This likely blowback should inhibit the motivation of terrorists to recreate the variola virus or enhance its pathogenicity.

## How Much Vaccine is Available, and How Should it Be Used?

The United States has ample supplies of second-generation and third-generation vaccines (*25*). The second-generation vaccine is ACAM2000 (Acambis, Cambridge, MA, USA), which is a plaque-purified distinct strain of New York City Board of Health vaccinia virus grown by using modern cell lines rather than the skin of calves. It produces reactions and immune responses similar to those of first-generation Dryvax vaccine (Wyeth Laboratories, New York, NY, USA) (*26*). Second-generation vaccines can be diluted 1:10 and still give excellent results. The third-generation vaccine is Immvamune (Bavarian Nordic, Kvistgaard, Denmark), a strain of modified vaccinia Ankara (MVA), which has been extensively tested for safety and protects animals from orthopoxvirus challenge (*1*). Two injections of MVA produce humoral and cellular immunity similar to that produced by first-generation and second-generation vaccines. Although a live virus, MVA does not replicate in human tissues and does not have the same risk of adverse reactions as first-generation and second-generation vaccines. It has been tested in patients with atopic dermatitis and in patients with HIV infections with T-cell counts >250 cells/mL (*1*).

Third-generation vaccines such as MVA may not be optimal for outbreak control. MVA is frozen, and thus must be thawed in the field (the manufacturer is developing a freeze-dried formulation). It requires syringes and needles and must be administered by someone trained to give injections. First-generation and second-generation vaccines are lyophilized and can be reconstituted and administered in the field with bifurcated needle scarification by persons with minimal training. Because MVA does not produce a visible lesion or scar, rapid determination of who has been already vaccinated is difficult. Optimal immunity with MVA requires 2 injections. In contrast, single injections are fully protective for first-generation and second-generation vaccines.

In the absence of a perfect animal model for smallpox, and because it is impossible to test these vaccines against smallpox, we have only laboratory evidence of their efficacy. Given these limitations, third-generation vaccines may be best for persons who anticipate possible exposure, such as military personnel or laboratory personnel working with orthopoxviruses. In an actual outbreak, ACAM2000 should be used for field vaccination. However, its use increases the risk for development of progressive vaccinia or eczema vaccinatum; these adverse events would then need to be treated with ST-246.

WHO is planning on creating a modest real, and substantial virtual, stockpile of vaccines. There are ≈60 million doses of vaccine in this stockpile and plans to increase it to ≈200 million doses. How much of this stockpile should be second-generation or third-generation vaccines that may not yet have been licensed widely? What should be the rules for release of such vaccines from the stockpile? Widespread use of first-generation or second-generation smallpox vaccines in era of AIDS and iatrogenic immune suppression by cancer chemotherapy or for transplant surgery seems unlikely unless there are actual cases of smallpox. In that situation, we might respond by using first-generation and second-generation vaccines with proven efficacies.

## Should Existing Variola Viruses be Destroyed?

The WHO-approved research agenda with variola major virus has been largely fulfilled (*1**–**5*). Modern viral genetics may have rendered destruction of the 2 official remaining stores of the virus moot because the virus can be recreated with minimal technical difficulty (vide supra). The US Institute of Medicine has issued a report that outlines robust scientific arguments for retaining the stocks of live virus (*27*).

Discussions about destruction of the remaining variola major virus stocks should not be limited to the scientific points set forth in the US Institute of Medicine report (*27*). Ethical, political, and public relations issues would be involved in recreating the smallpox virus. Retaining existing stores of live variola major virus has similar ethical and political problems. Terrorists or rogue states that have other weapons of mass destruction might see our possession of smallpox virus as a justification for their own development of a bioterrorist arsenal. If known official stocks are destroyed, then we will know that any new cases of smallpox are the result of deliberate maligning activities. Thus, as long as Russia and the United States possess the virus, we have lost the moral high ground. The known stocks of the virus ought to be destroyed, as has been repeatedly requested by the World Health Assembly.

## References

[1] Alcami A, Moss B. Scientific review of variola virus research, 1999–2010. Smallpox vaccines. In: WHO Advisory Committee on Variola Virus Research. Report of the Twelfth Meeting; 2010 Nov 17–18; Geneva, Switzerland. Geneva: World Health Organization; 2010. p. 20 [cited 2011 Feb 24]. http://whqlibdoc.who.int/hq/2010/WHO_HSE_GAR_BDP_2010.5_eng.pdf

[2] Huggins JW, Tikunova N. Scientific review of variola virus research, 1999–2010. Antiviral drug development for smallpox treatment. In: WHO Advisory Committee on Variola Virus Research. Report of the Twelfth Meeting; 2010 Nov 17–18: Geneva, Switzerland. Geneva: World Health Organization; 2010. p. 25–6 [cited 2011 Feb 24]. http://whqlibdoc.who.int/hq/2010/WHO_HSE_GAR_BDP_2010.5_eng.pdf

[3] McFadden G, Evans D, Shchelkunov S, Damon I. Scientific review of variola virus research, 1999–2010. Variola genomics. In: WHO Advisory Committee on Variola Virus Research. Report of the Twelfth Meeting; 2010 Nov 17–18; Geneva, Switzerland. Geneva: World Health Organization; 2010. p. 22 [cited 2011 Feb 24]. http://whqlibdoc.who.int/hq/2010/WHO_HSE_GAR_BDP_2010.5_eng.pdf

[4] Jahrling PB. Scientific review of variola virus research, 1999–2010. Animal models and pathogenesis. In: WHO Advisory Committee on Variola Virus Research. Report of the Twelfth Meeting; 2010 Nov 17–18; Geneva, Switzerland. Geneva: World Health Organization; 2010. p. 24 [cited 2011 Feb 24]. http://whqlibdoc.who.int/hq/2010/WHO_HSE_GAR_BDP_2010.5_eng.pdf

[5] Damon I, Meyer H, Shchelkunov S. Scientific review of variola virus research, 1999–2010. Laboratory diagnostics. In: WHO Advisory Committee on Variola Virus Research. Report of the Twelfth Meeting; 2010 Nov 17–18; Geneva, Switzerland. Geneva: World Health Organization; 2010. p. 21 [cited 2011 Feb 24]. http://whqlibdoc.who.int/hq/2010/WHO_HSE_GAR_BDP_2010.5_eng.pdf

[6] Fenner F, Henderson DA, Arita I, Ladnyi ID. Smallpox and its eradication. Geneva: World Health Organization; 1988.

[7] Nishiura H. Smallpox during pregnancy and maternal outcomes. Emerg Infect Dis. 2006;12:1119–21. 16836830 10.3201/eid1207.051531 PMC3293446

[8] Rao AR, Prahlad I, Swaminathan M, Lakshmi A. Pregnancy and smallpox. J Indian Med Assoc. 1963;40:353–63. 13973041

[9] Williams obstetrics. 23rd ed. Cunningham F, Leveno K, Bloom S, Hauth J, editors. New York: McGraw Hill Medical; 2010.

[10] Dixon CW. Smallpox. Boston: Little, Brown and Company; 1962.

[11] Massoudi MS, Barker L, Schwartz B. Effectiveness of postexposure vaccination for the prevention of smallpox: results of a Delphi analysis. J Infect Dis. 2003;188:973–6. 10.1086/378357 14513416

[12] Mortimer PP. Can postexposure vaccination against smallpox succeed? Clin Infect Dis. 2003;36:622–9. 10.1086/374054 12594644

[13] Kennedy JS, Frey SE, Yan L, Rothman AL, Cruz J, Newman FK, Induction of human T cell–mediated immune responses after primary and secondary smallpox vaccination. J Infect Dis. 2004;190:1286–94. 10.1086/423848 15346340

[14] Yang G, Pevear DC, Davies MH, Collett MS, Bailey T, Rippen S, An orally bioavailable antipoxvirus compound (ST-246) inhibits extracellular virus formation and protects mice from lethal orthopoxvirus challenge. J Virol. 2005;79:13139–49. 10.1128/JVI.79.20.13139-13149.2005 16189015 PMC1235851

[15] Huggins J, Goff A, Hensley L, Mucker E, Shamblin J, Wlazlowski C, Nonhuman primates are protected from smallpox virus or monkeypox virus challenges by the antiviral drug ST‐246. Antimicrob Agents Chemother. 2009;53:2620–5. 10.1128/AAC.00021-09 19349521 PMC2687232

[16] Mack TM. Smallpox in Europe, 1950–1971. J Infect Dis. 1972;125:161–9. 10.1093/infdis/125.2.161 5007552

[17] Mack T. A different view of smallpox and vaccination. N Engl J Med. 2003;348:460–3. 10.1056/NEJMsb022994 12496354

[18] Siegel JD, Rhinehart E, Jackson M, Chiarello L. Healthcare Infection Control Practices Advisory Committee. Guideline for isolation precautions: preventing transmission of infectious agents in healthcare settings [cited 2010 Dec 9]. http://www.cdc.gov/ncidod/dhqp/pdf/isolation 2007.pdf 10.1016/j.ajic.2007.10.007 PMC7119119 18068815

[19] Landers SH. Why health care is going home. N Engl J Med. 2010;363:1690–1. 10.1056/NEJMp1000401 20961239

[20] McFadden G. Killing a killer: what next for smallpox? PLoS Pathog. 2010;6:e1000727. 10.1371/journal.ppat.1000727 20126444 PMC2813274

[21] Alibek K, Handelman S. Biohazard. New York: Random House; 1999.

[22] Jackson RJ, Ramsay AJ, Christensen CD, Beaton S, Hall DF, Ramshaw IA. Expression of mouse interleukin-4 by a recombinant ectromelia virus suppresses cytolytic responses and overcomes genetic resistance to mousepox. J Virol. 2001;75:1205–10. 10.1128/JVI.75.3.1205-1210.2001 11152493 PMC114026

[23] Skowronski DM, Astell C, Brunham RC, Low DE, Petric M, Roper RL, Severe acute respiratory syndrome (SARS): a year in review. Annu Rev Med. 2005;56:357–81. 10.1146/annurev.med.56.091103.134135 15660517

[24] Skowronski DM, Petric M, Daly P, Parker RA, Bryce E, Doyle PW, Coordinated response to SARS, Vancouver, Canada. Emerg Infect Dis. 2006;12:155–8. 16494736 10.3201/eid1201.050327 PMC3291383

[25] Centers for Disease Control and Prevention. Emergency preparedness and response. Questions about smallpox vaccine storage and distribution [cited 2010 Dec 9]. http://emergency.cdc.gov/agent/smallpox/faq/storage/asp

[26] Frey SE, Newman FK, Kennedy JS, Ennis F, Abate G, Hoft DF, Comparison of the safety and immunogenicity of ACAM1000, ACAM 2000 and Dryvax in healthy vaccinia‐naive adults. Vaccine. 2009;27:1637–44. 10.1016/j.vaccine.2008.11.079 19071184

[27] Institute of Medicine. Live variola virus: considerations for continuing research, 2009 [cited 2010 Dec 9]. http://www.iom.edu/Reports/2009/LiveVariolaVirusContinuingResearch.aspx

